# Personalizing exoskeleton assistance while walking in the real world

**DOI:** 10.1038/s41586-022-05191-1

**Published:** 2022-10-12

**Authors:** Patrick Slade, Mykel J. Kochenderfer, Scott L. Delp, Steven H. Collins

**Affiliations:** 1 Department of Mechanical Engineering, Stanford University, Stanford, CA USA; 2 Department of Bioengineering, Stanford University, Stanford, CA USA; 3 Department of Aeronautics and Astronautics, Stanford University, Stanford, CA USA

**Keywords:** Biomedical engineering, Translational research, Mechanical engineering

## Abstract

Personalized exoskeleton assistance provides users with the largest improvements in walking speed^1^ and energy economy^2–4^ but requires lengthy tests under unnatural laboratory conditions. Here we show that exoskeleton optimization can be performed rapidly and under real-world conditions. We designed a portable ankle exoskeleton based on insights from tests with a versatile laboratory testbed. We developed a data-driven method for optimizing exoskeleton assistance outdoors using wearable sensors and found that it was equally effective as laboratory methods, but identified optimal parameters four times faster. We performed real-world optimization using data collected during many short bouts of walking at varying speeds. Assistance optimized during one hour of naturalistic walking in a public setting increased self-selected speed by 9 ± 4% and reduced the energy used to travel a given distance by 17 ± 5% compared with normal shoes. This assistance reduced metabolic energy consumption by 23 ± 8% when participants walked on a treadmill at a standard speed of 1.5 m s^−1^. Human movements encode information that can be used to personalize assistive devices and enhance performance.

## Abstract (web summary)

A portable ankle exoskeleton uses a data-driven method and wearable sensors to adapt to the user as they walk in a natural setting.

## Main

Exoskeletons that assist leg movement show promise for enhancing personal mobility but have yet to provide real-world benefits. Millions of people have mobility impairments that make walking slower^5^ and more fatiguing^6^, while millions more people have occupations that require strenuous locomotion^7^. In research laboratories, exoskeletons can increase walking speed^1,8,9^ and reduce the energy required to walk^2–4,10–16^, but these benefits have not yet translated to real-world conditions^17^. Providing beneficial assistance in the real world is difficult for several reasons: the specialized equipment used to personalize assistance is not available outside the laboratory; unlike walking on a treadmill, everyday walking occurs in many bouts of varying speed and duration; and devices must be self-contained and easy to use. In this study, we addressed each of these challenges to demonstrate effective exoskeleton assistance under naturalistic conditions.

Maximizing the benefits of exoskeleton assistance requires personalization to individual needs, which is challenging outside of a laboratory. The largest improvements in human walking performance have been achieved by individualizing assistance using human-in-the-loop optimization^1–4^, a process in which device control is systematically tuned to improve human performance while a person uses a device. Measuring important aspects of performance, including metabolic rate^16^, has required expensive laboratory equipment and long periods of steady treadmill walking^18^. Individualizing consumer or medical devices in this way would require several long visits to a specialized clinic, which would be costly and impractical. If human performance could instead be estimated quickly, using low-cost wearable sensors, optimization could be performed as people moved naturally through their daily lives. This might be possible using musculoskeletal modelling^19^, but such simulations are computationally intensive^20^ and require individualization. Data-driven models may be able to capture important features of human performance more simply^21–25^.

**Fig. 1:**
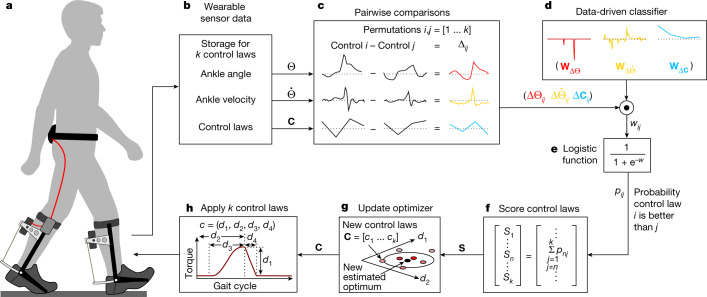
Data-driven exoskeleton optimization. We used data from laboratory tests to train a model that can perform optimization in real-time outside the laboratory. **a**, During optimization, the participant walks with the exoskeleton and experiences a sequence of *k* control laws, each defining a pattern of exoskeleton torque. The optimizer’s goal is to identify the torque pattern that maximizes performance. **b**, Ankle angle (θ) and ankle velocity ($\dot{\theta }$) for each stride are recorded from sensors on the exoskeleton. **c**, All possible pairs of control laws are then compared (*C*). For each pair, differences in segmented motion data (*Δ*) are calculated by subtraction. **d**, Differences in motion are multiplied with classifier model weights (*W*), using a dot product operation, to obtain the pair coefficient (*w*_*ij*_). **e**, A logistic function uses the pair coefficient to compute the probability (*p*_*ij*_) that the first control law is more beneficial than the second. **f**, The score (*S*) for each control law (*n*) is computed by summing the probabilities of all pairs that include that control law. **g**, Control laws are then ranked by score and used to update an optimizer. **h**, The optimizer selects a set of *k* new control laws, consisting of *d* parameters, to evaluate. This optimization process is repeated until convergence criteria are satisfied, in this case a set number of evaluations having been completed. During real-world experiments, optimization was performed on the exoskeleton’s microcontroller.

**Extended Data Fig. 1:**
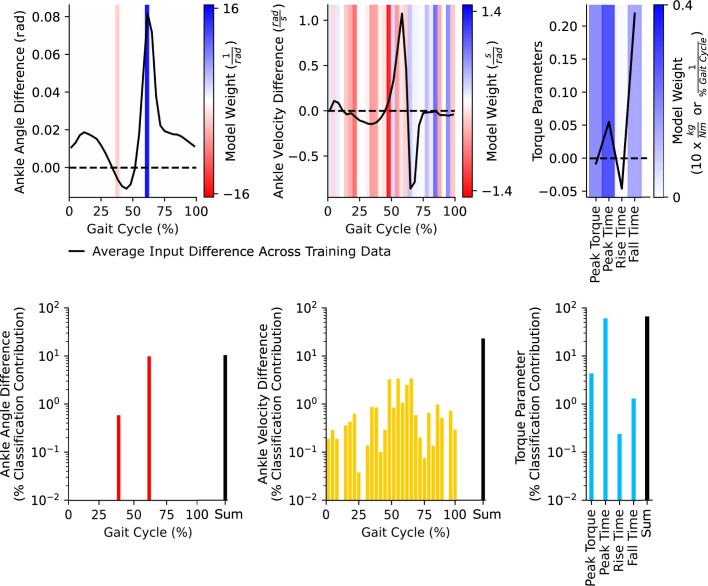
Weights, inputs, and effects for the data-driven classification model. The data-driven classifier decoded latent information from human movement that was not otherwise interpretable, allowing exoskeleton assistance to be optimized without laboratory-based measurement equipment. *Top row:* Model weights and mean inputs. The model compares data from two control laws at a time and associates inputs with higher or lower metabolic rate to estimate which control law resulted in a lower metabolic rate. Inputs comprised differences in ankle angle and ankle angular velocity at 30 different points in the gait cycle and differences in the four control law parameters of peak torque magnitude, peak time, rise time, and fall time. The data-driven model weights that multiply these differences are shown as a background colour of blue or red. Blue indicates that a positive difference is associated with lower metabolic rate, while red indicates that a positive difference is associated with higher metabolic rate. Darker colours indicate greater influence. Black lines depict the average, across all training data, of the differences in inputs. To generate this average, we ordered each pair-wise comparison by metabolic rate, such that inputs from the control law with a higher metabolic rate were always subtracted from those with a lower metabolic rate. Typical values of the model inputs differ, in part because of differences in units, and so the magnitudes of model weights do not correspond well to the contributions of those terms to the classification overall. *Bottom row:* The classification contributions of each term in the model, averaged over the entire training set. The percent contribution is calculated as the absolute value of the product of the model weight and the input difference, summed over all pair-wise comparisons, divided by the sum over all model terms. For the x-axes, 0% and 100% of the gait cycle refer to the instant of heel strike of the assisted limb at the beginning and end of one stride. Toe-off occurs at about 62% of the gait cycle. For a discussion of the intuitive meaning of the weights and contributions, please see the Methods subsection “Data-driven optimization”. Source data

We developed a data-driven model that relates human motion during exoskeleton-assisted walking to metabolic energy consumption and can be used outside the laboratory. Human movement arises from the interaction between the inertia of our body segments and forces from the environment and our muscles. We hypothesized that careful analysis could extract meaningful information about muscular energy expenditure from subtle changes in motion. In a previous experiment^4^, participants walked with exoskeleton assistance in about 3,600 different conditions while data were recorded from both laboratory equipment that measure biomechanical outcomes and low-cost, portable sensors on the exoskeleton. We trained a logistic regression model using this previous dataset (Extended Data Fig. 1). The data-driven classification model compared sensor data from two different patterns of exoskeleton assistance, each defined by a ‘control law’, and classified which control law provided a larger benefit. The model inputs were ankle angle and ankle velocity, segmented by gait cycle, and the torque parameters for each control law. The model then estimated the likelihood that the first control law resulted in lower metabolic energy expenditure. In essence, the classifier favoured later, larger exoskeleton torques and smooth, well timed movements that led to increased ankle extension at toe-off. During optimization, the user experienced a set of control laws, the data-driven model compared all possible pairs of control laws, the control laws were ranked, and an optimization algorithm^26^ updated the estimate of the optimal parameters and generated a new set of control laws to evaluate (Fig. 1). This process was repeated until convergence criteria were met.

**Fig. 2:**
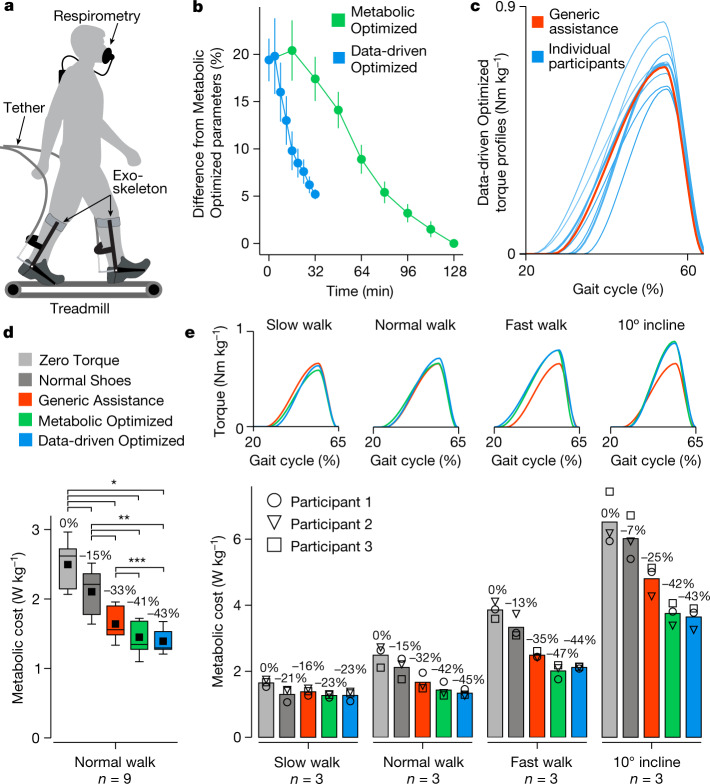
Data-driven optimization results. **a**, Exoskeleton assistance was applied using a tethered ankle exoskeleton emulator^43^. **b**, Assistance parameters optimized using the data-driven method converged to within 5% of the parameters identified using metabolic optimization, but in one-quarter of the time (*n* = 9). The error bars represent the standard deviation. **c**, Individual participants had unique Data-driven Optimized parameters, centred around the Generic assistance parameters. **d**, Data-driven Optimized assistance and Metabolic Optimized assistance resulted in similar metabolic costs of walking, significantly lower than with Zero Torque, Normal Shoes or Generic assistance, when walking at 1.25 m s^−1^ (ANOVA, *n* = 9, *P ≤ 2.7 × 10^−8^, **P ≤ 2.4 × 10^−5^, ***P ≤ 0.047). The boxes extend from the lower to upper quartile values of the data, with a line at the median and a dot at the mean. The whiskers extend between the minimum and maximum of the data values. **e**, Optimized torque patterns varied with walking condition, with similar changes in Data-driven Optimized and Metabolic Optimized parameters. Data-driven Optimized assistance and Metabolic Optimized assistance led to similar reductions in metabolic rate when walking at 0.75 m s^−1^ (slow), 1.25 m s^−1^ (normal) and 1.75 m s^−1^ (fast), and on a 10° incline at 1.25 m s^−1^. Source data

Data-driven optimization can use the information embedded in our movements to identify exoskeleton assistance patterns that are as effective as those found with laboratory-based methods, but in one-quarter of the time. We conducted experiments to optimize assistance with a tethered exoskeleton emulator (Fig. 2a). The data-driven optimization evaluated eight sets of control laws in 32 min, four times faster than the state-of-the-art approach using indirect respirometry to measure metabolic rate^2^ (Fig. 2b). Data-driven and metabolic optimization approaches identified the same participant-specific adjustments to assistance (Fig. 2c). Data-driven Optimized assistance and Metabolic Optimized assistance resulted in similar metabolic cost, which was significantly lower than the metabolic cost of walking with the exoskeleton in a Zero Torque mode (Fig. 2d). The average of the Data-driven Optimized parameters matched those of Generic assistance, which were taken from the best previous study^4^, but Data-driven Optimized assistance provided a larger benefit. This demonstrates the importance of individualization; even subtle changes in torque can lead to substantial performance enhancements. To test the generality of the data-driven model, we conducted experiments at a range of additional speeds and inclines with a subset of participants. The Data-driven Optimized assistance and Metabolic Optimized assistance resulted in similar torque profiles and metabolic cost reductions across these conditions (Fig. 2e). This shows that the data-driven classification model captured a fundamental relationship between exoskeleton torque, ankle movement and whole-body walking effort. The model approximates this biological relationship, precluding statistical guarantees of optimality. Nevertheless, our results demonstrate that human movement encodes information related to underlying physiological processes, and that data-driven methods can extract this information without laboratory equipment or complex multi-scale models.

**Fig. 3:**
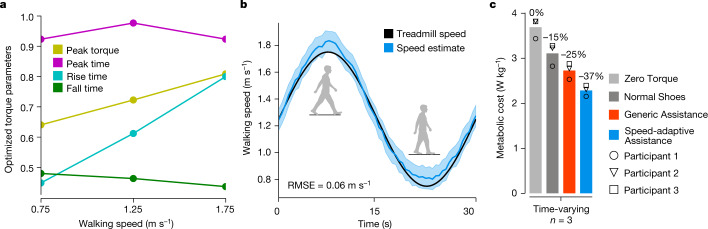
Speed-adaptive control. **a**, The Speed-adaptive controller interpolated between previously optimized assistance parameters to estimate the optimal parameters for each step based on walking speed on the previous step. Optimized values are normalized to the allowable range for each parameter. **b**, Ground truth and estimated walking speed for a representative participant. Speed was estimated on each step using a model that took stride period as an input (Extended Data Fig. 2) with a root-mean-square error (RMSE) of 0.06 m s^−1^. The shaded region represents the mean ± one standard deviation. **c**, When participants (*n* = 3) walked on a treadmill that varied speed sinusoidally between 0.75 m s^−1^ and 1.75 m s^−1^, Speed-adaptive assistance reduced the metabolic cost of walking more than the fixed Generic assistance. Source data

**Extended Data Fig. 2:**
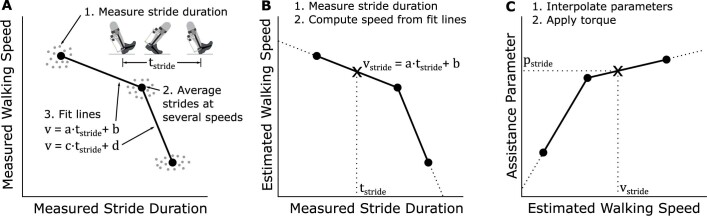
Speed-adaptive control approach. **a**, To calibrate the walking speed estimator, data are collected while the participant walks at several prescribed speeds, each within the range of speeds associated with a set of assistance parameters to be optimized. The measured stride durations and ground-truth speed measurements from those tests are used to fit an affine equation with linear regression. **b**, The resulting model can then be used to estimate walking speed based on measurements of stride duration alone. **c**, The speed-adaptive controller relates estimated walking speed to exoskeleton assistance parameters by interpolating between assistance parameters specified at a set of chosen speeds. In this case, there are three sets of optimized parameters corresponding to three different walking speeds.

We developed a speed-adaptive controller to adjust assistance based on natural variations in walking speed. People vary their walking speed widely during the day^27^ in response to changes in context^28^ and constraints^29^. Variations in speed complicate exoskeleton control and may help explain why assistive devices that reduce walking effort during steady walking on a treadmill have not provided similar benefits during use under more natural conditions^17^. The speed-adaptive controller we developed (Extended Data Fig. 2) interpolated between assistance parameter values previously optimized at different walking speeds (Fig. 3a) based on the estimated speed of each step (Fig. 3b). We tested Speed-adaptive assistance on a subset of participants as they walked on a treadmill with sinusoidally varying speeds. Speed-adaptive assistance reduced the energetic cost of walking more than Generic assistance with constant parameters (Fig. 3c). Adjusting exoskeleton assistance based on speed is an effective strategy for handling speed variations that occur during normal walking.

We created an untethered exoskeleton for real-world assistance using a design approach based on emulation and optimization. Wearable robotic devices are typically designed using models or intuition, built as specialized prototypes, and then tested. However, humans are highly complex and diverse, making it difficult to predict the range of characteristics that will be optimal across a population. As a result, most devices designed this way are unable to provide optimal assistance and often provide no benefit at all. To develop the untethered exoskeleton used in this study, we first performed experiments with versatile exoskeleton emulators^30^. These laboratory-based, tethered hardware systems allowed us to perform a wide range of control and optimization experiments (Figs. 1–3) and identify the electromechanical characteristics that our untethered device would need. Using these design guidelines, we built a specialized, untethered device that provides predictable, meaningful benefits. This emulation and optimization design paradigm can reduce the cost and time required to develop new wearable robots.

**Fig. 4:**
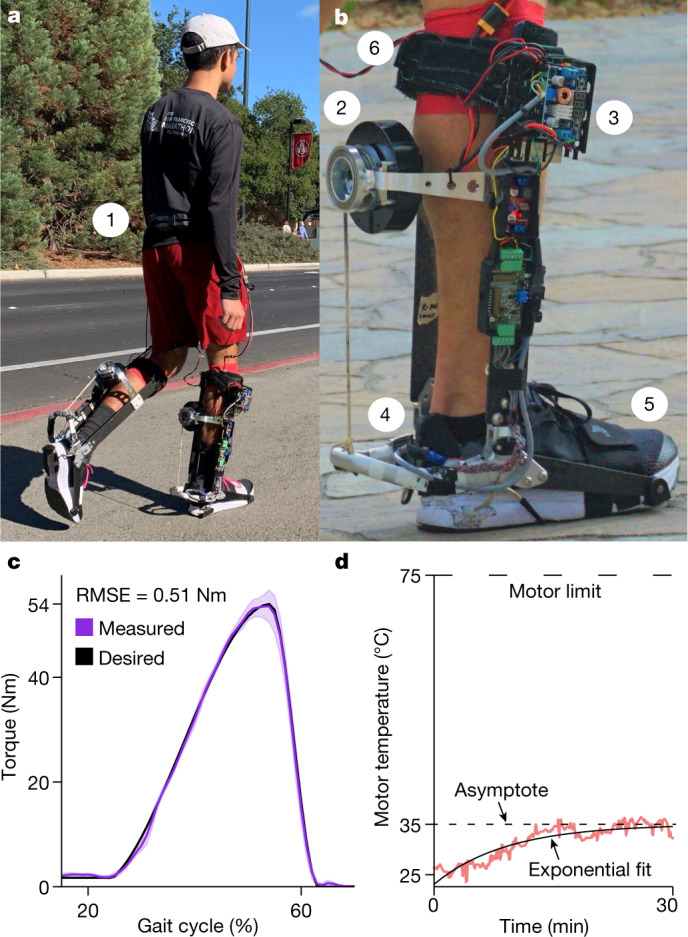
Untethered ankle exoskeleton. **a**, A participant walking in a community setting wearing the exoskeleton. **b**, The exoskeleton consists of (1) a battery pack worn on the waist, (2) a motor, drum and rope transmission to produce assistive torques, (3) electronics to receive sensor data, command the motor and perform optimization, (4) a carbon fibre and aluminium frame to transmit forces, and (5) a shoe and (6) a calf strap to transfer forces to the body. **c**, The motor can apply a peak torque of 54 Nm when walking at 1.5 m s^−1^, sufficient to match the optimized assistance parameters identified in emulator experiments. Torques were tracked accurately; the shaded region represents the mean ± one standard deviation. **d**, The motor temperature during 30 min of walking with maximum assistance remained well below the 75 °C thermal limit. An exponential fit indicated a steady-state temperature of 35.4 °C. Source data

**Extended Data Fig. 3:**
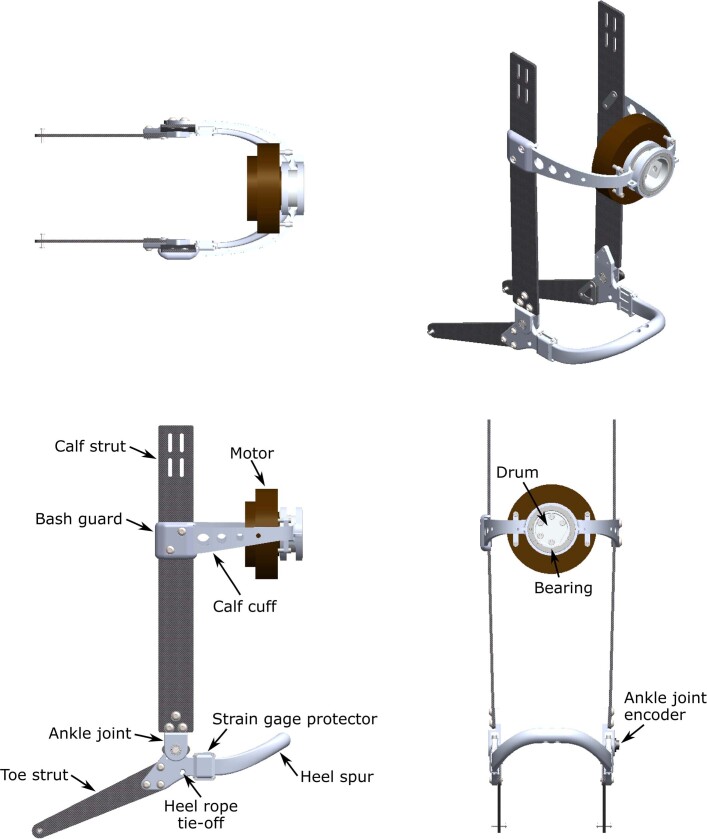
Diagram of untethered exoskeleton electromechanical hardware. These computer-aided design drawings depict the hardware elements of the untethered exoskeleton. The primary components are labelled. An image of the entire device, including textile components, can be found in Fig. 4b. A running shoe (not pictured) is attached to the toe strut with pins that extend from the tip of the toe strut into a carbon fiber plate embedded in the sole of the shoe. The heel of the running shoe is attached to the heel spur by a rope (not pictured) tied into holes on either side of the heel spur and passing through a plastic tube embedded in the sole of the shoe. A Vectran transmission cable (not pictured) transmits force from the drum to the tip of the heel spur. At the top of the calf strut, Velcro straps (not pictured) are connected to the strut through slots. These straps adhere to a separate Velcro strap (not pictured) worn on the shank of the leg, just below the knee. A complete bill of materials and set of computer-aided design files for this untethered exoskeleton assembly is included as Supplementary Data 2.

On the basis of the results of our emulator experiments, we designed a specialized, untethered ankle exoskeleton. The system consisted of an exoskeleton worn on each ankle and a battery pack at the waist (Fig. 4a and Supplementary Video 1). The exoskeleton was designed to apply the range of optimal torque profiles identified in the tethered optimization study (Fig. 2) while having low mass (1.2 kg per ankle). A brushless motor and custom drum transmission applied torque about the ankle joint, while portable electronics sensed the user’s motion and performed real-time control and optimization (Fig. 4b and Extended Data Fig. 3). The exoskeleton provided a peak torque of 54 Nm (Fig. 4c), which was about 50% to 75% of the biological ankle torque of participants in this study^31^. Torque was controlled using a mixture of classical feedback control and iterative learning^32^, with a tracking error of less than 1% of the peak torque. Maximum assistance could be applied continually without overheating the motor (Fig. 4d). The battery weighed 0.3 kg and powered the exoskeleton for at least 30 min on a single charge. While the energy cost of carrying mass near a distal joint is high^33^, locating motors and electronics near the assisted joint results in more efficient power transmission, a simpler design and lower total weight, which can yield large net benefits.

**Extended Data Fig. 4:**
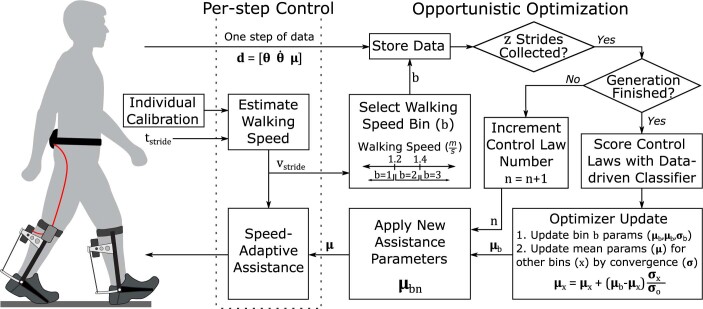
Optimizing assistance during real-world walking. The exoskeleton applied speed-adaptive control, which adjusted exoskeleton assistance parameters on each step. Stride duration (*t*_*stride*_) was used to estimate walking speed (*v*) as described in Fig. 3. While the participant walked, portable sensor data (*d*) were collected, which included ankle angle (*θ*), ankle velocity ($\dot{{\boldsymbol{\theta }}}$), and the control law defining exoskeleton assistance torque (*C*). If sufficient continuous strides (*z*) were not collected before the bout finished, the data were discarded and evaluation of the same control law began anew on the next walking bout. If sufficient continuous strides were collected, then data were stored for the associated control law number (*n*) and walking speed bin (*b*), selected based on the average walking speed for the collected strides. The control law number was incremented and the next control law was applied to the user. After six control laws had been applied for a given walking speed bin, forming one generation for the optimizer, the stored data were used to update the optimization parameters associated with that speed bin. When any bin performed an update, the estimate of the optimal parameter values (μ) for the other bins were also updated. Bins that were closer to convergence, indicated by a small value of the convergence parameter (σ) for that bin, were adjusted less. This approach allowed the optimizer to rapidly adapt to the participant early in the optimization, then to fine-tune the speed-specific parameters as the optimization progressed. Following the update, the optimizer selected a promising set of new control laws to be sequentially evaluated in the next generation for the associated walking speed bin.

We used the information encoded in a single walking step to optimize exoskeleton assistance while people walked naturally in short bouts of varying speed. People take thousands of steps per day, but real-world walking occurs in many separate bouts, most of which are short, with 90% being less than 100 steps in duration^34^. Speed is relatively consistent within each bout, but varies across bouts^27^. This fragmentation presents a challenge for collecting optimization data and efficiently fine-tuning assistance. Our data-driven optimization method addresses the problem of gathering useful data from short walking bouts by using kinematic data collected with every step. In pilot tests, we found that we could accurately compare control laws based on just 44 continuous steps, opportunistically captured during natural bouts, allowing our system to accumulate data from about 77% of steps in a typical day^34^. We addressed variations in speed by defining speed bins based on observed human behaviour, associating collected data with the appropriate bin, noting when sufficient data for any one speed bin had been accumulated, applying the data-driven classifier to rank assistance parameters and using these rankings to update the optimal parameter estimates for all speed bins (Extended Data Fig. 4).

**Fig. 5:**
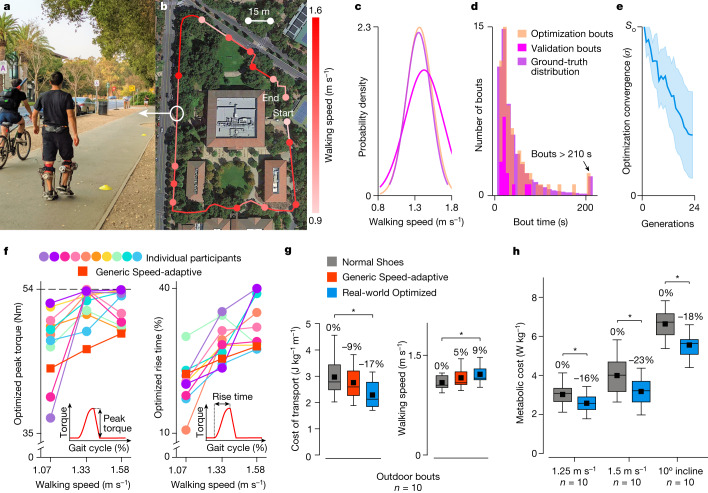
Real-world optimization of exoskeleton assistance. **a**, Participant walking on the public validation course. **b**, Map of the 566-m course used for optimization and validation. Participants walked the course repeatedly during optimization. **c**,**d**, Distribution of self-selected walking speeds (**c**) and walking bout durations (**d**) during optimization and validation, compared with previously recorded ground-truth distributions of real-world walking data^27,34^. **e**, As assistance was optimized over one hour of naturalistic bouts of walking, the convergence parameter (*σ*) continually improved. The error band represents one standard deviation. **f**, Optimized parameters for each participant were unique. The red squares depict the Generic Speed-adaptive assistance parameters, consisting of constant values for peak torque normalized to body mass (Nm kg^−1^) and rise time (percent gait cycle). For ease of comparison, we show the Generic Speed-adaptive peak torque in Nm, averaged across all participants. Peak torque values in this figure are not normalized to emphasize that several participants reached the maximum peak torque that the untethered exoskeleton could provide. The inset torque profiles indicate how each torque parameter affected the assistance profile. **g**, During validation under naturalistic walking conditions on the public course, Real-world Optimized assistance substantially reduced the energy cost of transport and increased walking speed compared with Normal Shoes (ANOVA, *n* = 10, *P ≤ 0.039). **h**, Real-world Optimized assistance also substantially reduced the metabolic cost of walking compared with Normal Shoes during benchmark treadmill conditions (ANOVA, *n* = 10, *P ≤ 0.023). Boxes extend from the lower to upper quartiles, with a line at the median and a dot at the mean. Whiskers extend between the minimum and maximum values. Source data

Real-world optimization quickly improved assistance during natural walking conditions. We conducted experiments in which participants performed one hour of walking in short bouts with exoskeleton assistance (Fig. 5a) on a public sidewalk (Fig. 5b and Supplementary Video 2). Participants were given ecologically relevant^35^ audio prompts^28^ that caused them to self-select walking speeds that matched a ground-truth distribution^27^ (Fig. 5c). Prompts were provided in random order and at specific intervals to obtain bout durations that also matched a ground-truth distribution^34^ (Fig. 5d). The optimizer steadily converged throughout the experiment (Fig. 5e), indicating steadily decreasing uncertainty as to which exoskeleton parameters would result in optimal performance according to the data-driven model. Post hoc analysis showed that the optimizer did not reach steady state, suggesting that additional time could have provided a better estimate of the optimal parameters. Peak torques optimized during naturalistic walking were larger than those from treadmill-based experiments (Fig. 5f). Participants may have felt more stable during outdoor walking^36^, allowing them to benefit from larger torques, consistent with observations from other comparisons of outdoor and treadmill walking with exoskeleton assistance^37^.

Real-world Optimized assistance increased self-selected walking speed and reduced the metabolic energy expended per distance travelled during naturalistic walking. In a separate validation experiment, participants performed a fixed set of outdoor walking bouts with varying durations and speeds, while ground-truth metabolic rate and speed were measured (Supplementary Video 3). Condition order was randomized (Extended Data Table 1). With Real-world Optimized assistance, the energetic cost of transport was reduced by 17 ± 5% (analysis of variance (ANOVA), *n* = 10, *P* = 0.039) and walking speed was increased by 9 ± 4% (ANOVA, *n* = 10, *P* = 0.031) compared with Normal Shoes (Fig. 5g). These energy savings are equivalent to removing a 9.2 kg backpack^38^, and the increase in walking speed of 0.12 m s^−1^ is similarly meaningful^39^. Real-world Optimized assistance provided roughly twice the benefits of Generic Speed-adaptive assistance, indicating that personalization was an important contributor to these benefits. Generic Speed-adaptive assistance may have provided a larger benefit if it had used the average of the torque parameters optimized during outdoor walking, rather than treadmill walking. These results demonstrate that lower-limb exoskeletons can provide meaningful benefits under naturalistic walking conditions and provide benchmarks for assessing the real-world benefits of future devices. Assistance can be personalized automatically in a natural setting, seamlessly improving human–robot interaction over time.

**Extended Data Fig. 5:**
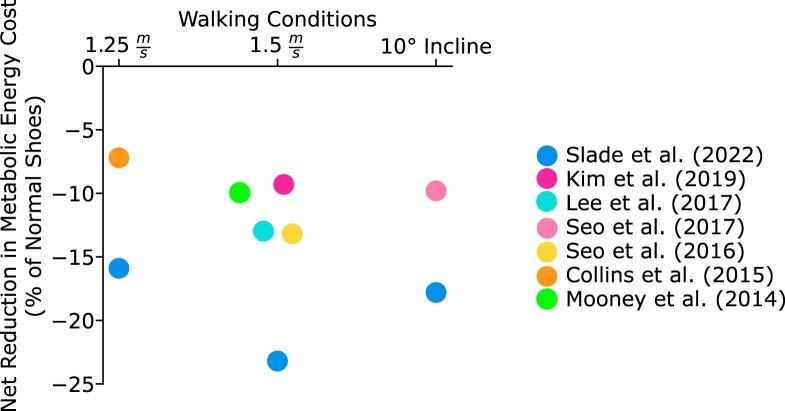
Exoskeleton comparison for standardized walking conditions. We compared the benefits of Real-world Optimized assistance from the untethered exoskeleton under standardized laboratory conditions to those of prior untethered exoskeletons^10–16^. We considered only the results of tests that: compared exoskeleton-assisted outcomes to walking in normal shoes without an exoskeleton; used standard indirect respirometry procedures; had sufficient sample sizes; and applied walking conditions within 10% of the chosen walking speeds and inclines in this study, which were chosen to allow comparison to the largest prior improvements in metabolic rate. For legibility, in this figure we depict only results within a 5% reduction in net metabolic cost of the best prior results for each category. Please see the Methods subsection “Comparison to other untethered exoskeletons” for a complete explanation of the methods used to select amongst prior exoskeleton experiments. Real-world Optimized assistance from the untethered exoskeleton resulted in large improvements in energy cost. Source data

**Extended Data Fig. 6:**
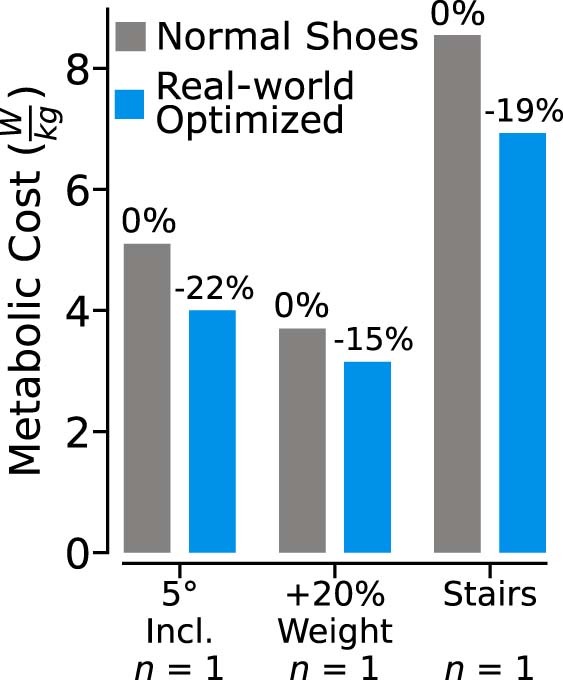
Additional untethered exoskeleton treadmill condition evaluations. For one pilot participant (*n* = 1), walking with Real-world Optimized assistance reduced the metabolic cost of walking compared to Normal Shoes during several additional treadmill conditions. These results suggest that Real-world Optimized assistance may perform well during a wide range of common walking activities. The conditions were walking at 1.25 m s^−1^ on a 5° incline, walking at 1.5 m s^−1^ while wearing a vest weighing approximately 20% body weight, and climbing stairs at a rate of 50 steps per minute. These results are not included in the main text due to their preliminary nature compared to the primary study outcomes. Source data

Assistance optimized under real-world conditions produced even larger benefits under standard treadmill conditions. After performing optimization in a public setting, we tested our untethered exoskeleton during standardized laboratory walking conditions to directly compare with previous devices^16^. Real-world Optimized assistance reduced the energy cost of treadmill walking by 16% at 1.25 m s^−1^, 23% at 1.5 m s^−1^, and 18% when walking up a 10° incline (ANOVA, *n* = 10, *P* < 0.023) compared with Normal Shoes (Fig. 5h and Extended Data Table 2), approximately twice the benefits of the previous devices with the best performance for these conditions (Extended Data Fig. 5). The energy savings during inclined walking were equivalent to removing a 15.2 kg backpack^40^. Pilot results suggest that the device provides similar benefits under other conditions, including walking on a 5° incline, loaded walking and stair climbing (Extended Data Fig. 6). Emulator-informed hardware design coupled with opportunistic, data-driven optimization led to exceptional performance enhancements across walking conditions.

Participants reported that the untethered exoskeleton was easy to use and relatively comfortable. Wearable robotic devices should be usable, comfortable and functional for everyday activities to be adopted by users^41^. Participants reported that the exoskeleton was relatively easy to use (Extended Data Table 3), ranking it in the 65th percentile of previously surveyed consumer devices^42^. Participants found that the exoskeleton did not interfere with their clothing and had a manageable weight, but were neutral as to whether it would be comfortable to wear throughout the day (Extended Data Table 4). Participants reported that it was easy to put on and take off the exoskeleton, stand while wearing the exoskeleton, and walk indoors and outdoors for extended periods with the exoskeleton (Extended Data Table 5). Six of the ten participants reported that they would prefer using the exoskeleton rather than normal shoes if they were to walk the public course again. The device we tested is a research prototype and not a refined product; substantial improvements would be required to allow reliable, unsupervised use during typical daily activities. The survey results suggest that it may be possible to create mobility-enhancing products that are easy to use, comfortable and reliable, and that many people may opt to use them.

These approaches to real-world personalization, adaptive assistance and specialized exoskeleton design could potentially be extended to address the needs of workers with physically demanding jobs and people with mobility impairments. A similar overall development approach could be used to address the most important outcomes for each biomechanically and neurologically similar group. Assistance could aid a variety of tasks, such as stair climbing or lifting, and improve other aspects of performance, such as balance or joint pain. In each case, additional training data could be collected in the laboratory and used to train new data-driven models, illuminating the information contained within the body’s movements for each task. With each training dataset, the learned models could be made more capable, progressively building more general relationships between movement and performance outcomes. Data from laboratory-based emulation and optimization experiments could simultaneously provide design guidelines for products. When used regularly, we expect devices like this to become finely tuned to the needs of each individual, resulting in larger performance enhancements than observed in this study. Longitudinal experiments will be needed to understand how such assistance affects behaviour and quality of life; as moving becomes easier, we hope to find that people will be more active, helping them to lead healthier lives.

## Methods

### Experimental design

The research objective was to personalize exoskeleton assistance during real-world walking. To achieve this objective, we proposed a method of data-driven optimization, which uses portable sensors on the exoskeleton to personalize assistance for each participant. We hypothesized that Data-driven Optimized assistance would provide larger reductions in metabolic rate than Generic assistance. We conducted a power analysis based on previous laboratory-based optimization experiments and found that a sample size of eight participants was necessary for the planned validation experiments. This analysis used a power of 1 − *β* = 0.8, a significance level of *α* = 0.05, the difference in mean metabolic rate between optimized (1.44 W kg^−1^) and generic (1.64 W kg^−1^) assistance from a previous experiment^4^, and the variability in metabolic rate (standard deviation 0.15 W kg^−1^) from the same experiment^4^. In this context,* β *is the probability of incorrectly accepting the null hypothesis. We collected data from nine participants for the tethered exoskeleton experiments and ten participants for the untethered exoskeleton experiments. We tested more participants than the minimum number determined by the power analysis to provide a factor of safety in case data from any participants were found to be unusable during later analysis. All participants that were recruited completed the corresponding experiment, and all data from all participants were included in each corresponding analysis. All participants had at least 8 h of experience walking with assistance from powered ankle exoskeletons, minimizing the effects of training that can occur while participants learn to walk with an exoskeleton^4^. All participants were volunteers and provided written informed consent before completing the protocol (IRB-48749), which was approved by the Stanford University institutional review board. Consent was obtained for publication of identifiable images of research participants. The experiments consisted of human participant testing in both laboratory and outdoor settings. Participants wore bilateral ankle exoskeletons and walked under a series of assistance conditions in a randomized order. Each of the experiments is described in the following sections. We used a one-way ANOVA to determine whether differences in the metabolic cost of walking across assistance conditions were different from zero.

### Measuring the metabolic cost of walking

The metabolic cost of walking was computed with measurements from respirometry equipment. Respirometry equipment was used to measure the volume of carbon dioxide and oxygen exchanged on each breath. A standard equation was used to compute metabolic energy expenditure in watts for each breath^44^. Metabolics measurements during the real-world walking experiment and validation were collected with portable respirometry equipment worn using a vest on the participant’s torso (K5, COSMED). Metabolics measurements during other exoskeleton experiments were collected with tethered respirometry equipment (Quark CPET, COSMED). Metabolics data were recorded during a quiet standing condition at the beginning of each day of experiments. This quiet standing value was removed from subsequent measurements to isolate the energy cost associated with walking and remove any absolute error associated with respirometry system calibration. The change in metabolic rate as a percentage of a baseline condition, measured within the same experiment, is reported as the primary outcome to account for differences in respirometry equipment calibration coefficients between data collections. Participants refrained from all food and drink except for water for at least 3 h before experiments that included respirometry measurements to avoid confounds from the thermal effect of food. Steady-state metabolic cost was computed by averaging data from the last 3 min of each 6-min condition. Cumulative metabolic cost was computed as the total energy expended during the condition^45^, including the metabolic cost beyond that of quiet standing for 3 min following completion of the condition, following methods from previous studies of non-steady gait^23,29^. Excess oxygen consumption and carbon dioxide production during the return to steady state in quiet standing reflect delays between instantaneous energy use at muscles and expired gas measurements that arise owing to mitochondrial, transport and respiratory dynamics^18^. Including respiratory data from the period following activity enables more accurate measurement of the energy actually expended during short bouts of walking^46^. The energetic cost of transport was calculated as the cumulative metabolic cost divided by the total distance walked.

### Exoskeleton assistance conditions

A variety of exoskeleton assistance conditions were evaluated to determine the benefits that they provided to the user. These assistance conditions included walking in Normal Shoes and walking with the exoskeletons while they applied Zero Torque, Generic assistance, Speed-adaptive assistance, Generic Speed-adaptive assistance, Metabolic Optimized assistance, Data-driven Optimized assistance and Real-world Optimized assistance.

We tested walking in Normal Shoes, without the exoskeleton, as a baseline condition for the untethered exoskeleton experiments. Ideally, assistance from an untethered exoskeleton would lead to a lower metabolic cost than walking in Normal Shoes, providing a net benefit to the user. Separate pairs of the same type of Nike running shoe, weighing 0.3 kg per shoe, were used for the Normal Shoes condition and incorporated into the tethered exoskeleton and the untethered exoskeleton.

The Zero Torque mode was an exoskeleton condition in which the exoskeleton provided no assistive torques. During this mode, the exoskeleton maintained a small amount of slack in the cable transmission so that virtually no torque was applied to the ankle. This condition was used as a baseline for experiments with the tethered exoskeleton (but not for experiments with the untethered exoskeleton) because it allowed us to isolate the benefits of exoskeleton assistance from the energetic costs of wearing the emulator, which were expected to differ from those of an untethered device specialized to provide the same assistance.

The Generic assistance condition used a fixed set of assistance parameters identified from a previous optimization experiment. Generic assistance patterns have been found to reduce the metabolic cost of walking less than assistance personalized to each individual^2–4^. The tethered exoskeleton experiments in this study used Generic assistance computed by averaging the optimized parameters from a group of participants in a previous experiment using the same tethered ankle exoskeleton that had provided the largest energetic benefits owing to exoskeleton assistance so far^4^. The Generic assistance pattern allowed us to estimate the contributions of personalization through data-driven optimization in tests with the tethered emulator.

During Speed-adaptive assistance, the exoskeleton used estimates of walking speed to select assistance parameters expected to be more effective at that speed. The speed-adaptive control approach is described in detail in ‘Speed-adaptive controller and validation experiment’. In the Speed-adaptive condition, the controller interpolated between separate sets of assistance parameters that had been optimized for the same participant at walking speeds of 0.75 m s^−1^, 1.25 m s^−1^ and 1.75 m s^−1^. This condition was used to test the efficacy of the speed-adaptive control approach for handling speed variations during treadmill walking with the tethered exoskeleton emulator.

In the Generic Speed-adaptive assistance condition, the speed-adaptive controller selected assistance parameters expected to be more effective at that speed for an average participant. The controller interpolated between separate sets of Generic assistance parameters for each walking speed, computed by averaging the optimized assistance profiles from the tethered exoskeleton experiments in this study (Fig. 2). The generic parameters for 0.75 m s^−1^ and 1.75 m s^−1^ were computed by averaging across three participants’ optimized parameters, whereas the generic parameters for walking at 1.25 m s^−1^ were computed by averaging ten participant’s optimized parameters. The Generic Speed-adaptive assistance condition was compared with Real-world Optimized assistance during overground walking with the untethered exoskeleton. Ideally, the Generic Speed-adaptive parameters would have been taken as the average values from real-world optimization, but those values were not yet known at the time the experiment was conducted. The Generic Speed-adaptive condition provided the best available comparator for isolating the benefits of personalization during overground experiments with the untethered exoskeleton.

Metabolic Optimized assistance was personalized based on metabolic measurements using a previously established optimization method^2^ for identifying the exoskeleton control parameters that minimize the metabolic cost of walking for a specific person. To perform metabolic optimization, a participant walked on a treadmill while receiving exoskeleton assistance. The same assistance profile, or ‘control law’, was provided to the participant for 2 min while respirometry measurements were recorded and the steady-state metabolic cost of walking for that control law was estimated^18^. Participants repeated this process of walking on a treadmill for 2 min per control law until a fixed number of control laws had been evaluated. We refer to the set of control laws to be evaluated as one ‘generation’ of control laws—the terminology used in evolution-inspired optimization strategies such as the covariance matrix adaptation evolutionary strategy (CMA-ES)^26^. Each generation of control laws must be tested before updating the estimate of the optimal control law and generating a new generation of control laws to test. On the basis of heuristics relating generation size to the number of parameters to be optimized^26^, the lab-based experiments with four optimization parameters had participants complete eight control laws per generation. After each generation, an optimizer (CMA-ES) ranked the control laws in order of metabolic cost, updated the optimization parameters and selected a new set of promising control laws to evaluate. This optimization approach was established in previous experiments that demonstrated large improvements in the metabolic energy cost of walking and running with exoskeleton assistance^2,4,47^. This optimizer was selected because it is sample efficient, meaning that it requires relatively few evaluations to reach a reliable estimate of the optimal parameters. In the context of exoskeleton optimization, that means fewer exoskeleton control modes to be experimentally tested on the human participant, which is important both to study design and to real-world use of devices that personalize assistance. Metabolic Optimized assistance was used to validate the data-driven optimization approach in the first experiments with the tethered exoskeleton emulator.

Data-driven Optimized assistance was personalized using data-driven optimization. The data-driven optimization used the same optimization framework as the metabolic optimization, except that it used the data-driven classifier, rather than indirect respirometry measurements, to perform the ranking step. The classifier was trained on data from a previous laboratory experiment^4^ and compared control laws based on the exoskeleton torque parameters applied and the resulting ankle angle and ankle angular velocity (Fig. 1 and Extended Data Fig. 1). The data-driven optimization condition was applied in tests of the data-driven optimization approach using the tethered exoskeleton emulator.

Real-world Optimized assistance used speed-adaptive control with parameters that were personalized using the data-driven optimization approach while walking with the untethered ankle exoskeleton under naturalistic conditions. The controller used the same speed estimation and adaptation approach as with the Generic Speed-adaptive condition, except that the parameters for each speed were personalized to the individual participant using opportunistic optimization. The Real-world Optimized parameters were computed using the approach detailed in ‘Opportunistic optimization approach’. The Real-world Optimized condition was applied in outdoor and treadmill tests with the untethered exoskeleton.

### Data-driven optimization

Data-driven optimization personalized assistance using a data-driven classification model to determine which exoskeleton control parameters provided the largest benefits for each person. The participant walked while receiving a sequence of different patterns of exoskeleton assistance, each defined by the corresponding control law. During laboratory-based experiments, participants walked on a treadmill for 30 s for each control law. During real-world experiments, participants walked overground for 44 continuous steps for each control law. A fixed number of exoskeleton control laws, comprising one generation of the evolution-inspired optimizer, were then ranked using the data-driven classifier. The optimizer then updated its estimate of the optimal parameters and generated a new set of control laws to evaluate. The following paragraphs detail what type of data were collected, how the data were processed, how the data-driven classification model evaluated control laws, how the optimizer was updated based on the data-driven classifications and how a new set of control laws was selected for evaluation in the next generation of optimization (Fig. 1).

Exoskeleton torque control parameters, defined by the control law, were fixed within each evaluation period. The person experienced several control laws before the data-driven model processed data and the optimizer updated its estimate of the optimal assistance parameters and generated a new set of control laws to evaluate.

The data-driven model input consisted of carefully processed portable sensor data, including ankle angle and ankle velocity measurements and the control law parameters that set the pattern of exoskeleton torque. The angle and velocity measurements were sampled using an absolute rotary encoder at the ankle joint of the exoskeleton worn on the left leg. The control law parameters consisted of four values: peak torque magnitude, peak time, rise time and fall time^2^.

Portable sensor data were processed by segmenting the ankle angle and velocity measurements by gait cycle and then discretizing the data for each gait cycle into a discrete number of bins. The gait cycles were segmented whenever a heel strike was detected by the pressure-sensing insoles. The first six gait cycles of data were discarded to avoid confounds from fast adaptation^48^ by the person in response to the new assistance pattern—in pilot tests, we found that data from these first six steps exhibited substantial changes in ankle kinematics, while subsequent strides were more consistent. The remaining gait cycles were discretized by averaging the measurements within each of 30 discrete bins and then averaging each bin across the gait cycles for that control law. The processed data were reshaped into a single vector with 64 values: 30 binned values for the ankle angle across the gait cycle, and 30 binned values for the ankle velocity across the gait cycle, and 4 values for the torque parameters. The model input consisted of the vector of data for one control law subtracted from the vector of data for a different control law, which also comprised 64 values. This difference in the sensor measurements provided the model with information about how the person’s movements and exoskeleton torque differed between the two control laws. The choice to segment data by gait cycle follows our previous findings that data-driven models can more accurately estimate metabolic energy expenditure from sensors worn by unassisted humans when the data are formatted in this way^23^.

The data-driven classification model was trained to compare two control laws at a time, determining which control law was estimated to have provided a larger reduction in the metabolic cost of walking. The data-driven classification model was a logistic regression model. To train the data-driven classifier, we input previously collected data^4^ that included portable sensor data, in the form of exoskeleton joint angles and velocities, and ground-truth labels, in the form of metabolic measurements, for many exoskeleton control laws. The sensor data were taken as input into the model to estimate the likelihood that the first of the compared control laws resulted in a lower metabolic cost of walking compared with the second control law. The resulting probability was a continuous value from 0 to 1, with 1 indicating the highest likelihood that the first control law reduced the metabolic cost of walking more than the second control law. The ground-truth labels were computed by subtracting the measured metabolic costs, estimated from 2 min of respirometry data, for the two control laws. A label with a negative value indicated that the first control law was more beneficial, meaning that it reduced the metabolic cost of walking more than the second control law. A positive-valued label indicated that the second control law was more beneficial. The previously collected training data were from an experiment in which 10 participants walked under approximately 3,600 different exoskeleton control laws^4^. When training the data-driven classifier, we used regularization, a technique that encourages simpler models and avoids overfitting to training data, to improve model estimates for new data points that were not in the training set. In this case, we used a lasso regularization term that penalized the absolute value of the model weights multiplied by a regularization parameter with a value of 1.

The data-driven classification model was trained to capture a relationship between leg movement, exoskeleton torque parameters and the metabolic cost of walking with assistance. The linear weights used by the data-driven classifier are visualized using a colour code in Extended Data Fig. 1. To aid interpretation, we also overlay the mean difference for each model input as a black line. This was calculated as the value from the control law resulting in lower metabolic rate minus the value from the control law resulting in higher metabolic rate, averaged across all pair-wise comparisons, such that the sign of the mean difference is meaningful. We also provide the cumulative contributions of each term in the model to classification over the entire training set. The percent contributions are calculated as the absolute value of the product of the model weight and the difference input, summed over all pair-wise comparisons, divided by the sum over all model terms. Even linear data-driven models can be difficult to interpret because of the complex interactions between model terms through the dynamics of the underlying system, which can be nonlinear and coupled. In this case, the underlying system is a human walking with an exoskeleton, and we expect strong interactions between exoskeleton torques, joint velocities and joint angles, and between states at different times in the gait cycle. The model may be capturing aspects of these interactions in non-obvious ways. Nevertheless, we can gain some intuition about the relationships that the model may have identified if we consider the effects of key model terms independently.

The model weights associated with differences in ankle kinematics suggest that a lower metabolic rate was associated with increased ankle plantarflexion at toe-off, while guarding against premature onset of push-off, excessive plantarflexion velocity and reduced dorsiflexion mid-stance. The largest single contributor to classification based on ankle kinematics, constituting about 10% of the total, favoured a larger plantarflexion angle at 62% stride, the time of toe-off during normal walking. A large negative weight on the difference in ankle velocity at 48% stride seemed to penalize conditions that resulted in premature onset of ankle push-off. A sequence of negative weights on ankle plantarflexion velocity during push-off seemed to favour slower, smoother movement during that phase. Taken together, these velocity regulation terms constituted about 16% of the total classification. Smaller negative weights on ankle angle at 38% stride and ankle velocity before the onset of push-off suggest a preference for conditions with greater mid-stance dorsiflexion. The model terms associated with ankle angle and velocity were most informative during late stance, when the concentric contractions of the plantarflexor muscles are less efficient and exoskeleton torque may have the most capacity to reduce metabolic cost^49,50^. During the leg swing phase, model terms contributed little to the total classification, consistent with expectations for an ankle exoskeleton that produced no torque when the foot was off the ground. The sum of all model weights associated with ankle angle and velocity were 10% and 23% of the total, respectively.

The model weights on differences in torque parameter values indicated that lower metabolic rate was associated with a later time of peak torque and, to a lesser extent, a larger peak torque magnitude. Exoskeleton assistance was governed by a torque pattern defined by four parameters: peak torque magnitude, peak time, rise time and fall time^2^. These four parameters had allowable ranges of 0 to 1 Nm kg^−1^, 40% to 55% stride, 20% to 40% stride, and 10% to 20% stride, respectively. Fall time was further constrained to be at most equal to the difference between peak time and the time of toe-off, which prevented application of torque during the swing phase. The largest single contributor to classification based on exoskeleton torque, constituting about 60% of the total, favoured applying peak torque at a later time in the gait cycle. As the peak time was constrained, this term had the effect of maintaining a peak time close to the upper limit of 55% stride. Consequently, fall time was effectively constrained to its lower bound of 10% stride. The large model weight on peak time is consistent with previous observations that the timing of ankle exoskeleton assistance is important^51^, and that later onset of torque assistance can correspond to larger improvements in metabolic rate^52^. The data-driven model also favoured larger peak torque magnitudes, with the associated term contributing about 4% to the total classification. Interestingly, peak torque was not driven to its upper limit for most participants or conditions, and the classification contribution of this term was about ten times less than the sum of contributions from ankle kinematics. This suggests that how a person reacts to exoskeleton assistance is more important for determining metabolic rate than the magnitude of the torque and power provided by the exoskeleton.

Exoskeleton control laws were ranked using the probability values estimated by the data-driven classifier. Each pair of control laws that were passed to the data-driven classification model yielded one probability value, defining whether the first control law was expected to have provided a larger benefit than the second control law. All possible pairs of control laws were classified with the data-driven model to obtain a complete set of probability values. Each control law was scored by summing the probabilities from all pairs that included that control law. The control laws were ranked by the magnitudes of their scores, with a larger value indicating that the control law was more likely to provide a larger reduction in the metabolic cost of walking (Fig. 1). This ranking step replaced the previous approach based on metabolic measurements from indirect respirometry equipment, allowing optimization to take place outside the laboratory using inexpensive sensors and a microcontroller on the exoskeleton. Using the data-driven ranking, the optimizer then updated its internal parameters and generated a new set of control laws to evaluate. The new estimate of the optimal control law was equal to the weighted average of the best-performing control laws. New control laws were selected from a distribution around this estimate of the optimum, with the shape of the distribution set by the covariance matrix and the spread of the distribution set by the convergence parameter.

The data-driven optimization process can be better understood by working through these steps using example data. Imagine that we have three control laws, labelled 1, 2, and 3, which happen to be in order of increasing metabolic cost and decreasing peak torque. Imagine that these control laws had identical torque timing parameters and resulted in identical ankle angles and ankle velocities. When performing the three pair-wise comparisons (Fig. 1c), the differences between ankle angle and ankle velocity would be zero. The differences between torque parameters (Δ*C*_12_, Δ*C*_13_ and Δ*C*_23_) would each be a vector with one positive value followed by three zeros. When taking the dot product of the parameter differences with the model weights on control parameters, which are all positive (Fig. 1d), the pair coefficients (*w*_12_, *w*_13_, and *w*_23_) would all be positive scalars. For each of these pair coefficients, the logistic function (Fig. 1e) would return a probability greater than 0.5, indicating that the first control law in the pair is likely to have a lower metabolic cost than the second control law. Let us imagine that each of the probability values (*p*_12_, *p*_13_, and *p*_23_) was 0.9. When performing the control law scoring step (Fig. 1f), the score for the first control law (*s*_1_) would be the sum of *p*_12_ and *p*_13_, or 1.8. The score for the second control law (*s*_2_) would be the sum of *p*_21_ and *p*_23_. As *p*_21_ is the complement of *p*_12_, *p*_21_ = 1 – *p*_12_ = 0.1. Thus, *s*_2_ would equate to 1. The score for the third control law (*s*_3_) would be the sum of *p*_31_ and *p*_32_, or 0.2. Thus, the scores would correctly rank the control laws in terms of metabolic cost. The optimizer would then use this ranking to perform an update, estimating that the optimal torque parameters were close to those of control law 1, but slightly offset towards control law 2. The optimizer would then select new control laws to evaluate, drawn from a distribution around the new estimate of the optimal parameters (Fig. 1g).

### Tethered optimization experiments

To compare the efficacy of data-driven optimization to a range of other assistance conditions, we conducted tethered exoskeleton experiments in an indoor laboratory setting. Participants wore tethered bilateral ankle exoskeleton emulators^43^. Exoskeleton assistance was governed by a torque pattern characterized by four parameters: peak torque magnitude, peak time, rise time and fall time^2^. The exoskeleton control loop ran at 1,000 Hz on a real-time computer (Speedgoat). Exoskeleton sensor measurements were recorded at a rate of 2,000 Hz, including pressure values from shoe insoles, commanded torque parameters, measured torque, ankle angle, and ankle velocity. Measurements were used to estimate time within the gait cycle as a percentage of the total gait cycle time, which was used to calculate the desired torque. Torque tracking was accomplished using a combination of classical feedback control and iterative learning, which accounted for errors that consistently occurred at the same point in the gait cycle on each step^30^.

Two tethered exoskeleton experiments were used to evaluate the effectiveness of various assistance conditions. The first experiment compared assistance conditions while participants walked at 1.25 m s^−1^, a normal walking speed previously used for metabolic optimization experiments^2,4^. Healthy young adults (*n* = 9, 5 men and 4 women; age, 24.8 ± 1.8 yr; body mass, 65.3 ± 8.0 kg; height, 1.73 ± 0.07 m) completed a two-day experimental protocol. On the first day, participants performed experiments to personalize assistance parameters with metabolic optimization and data-driven optimization, in a randomized order. Participants completed eight generations of optimization for each approach. Each generation consisted of eight control laws. The optimizations were initialized with the Generic assistance parameters, corresponding to the average of the optimized parameters identified for a previous group of expert participants^4^. The optimizations were initialized with the covariance matrix set to the identity matrix and a scaling factor that corresponded to 20% of the range of the normalized assistance parameters (a sigma value of 0.1). The metabolic optimization control laws lasted 2 min, which allowed steady-state metabolic cost to be estimated from respirometry data with a good balance between the time required for each control law and estimation accuracy^2^. This led to a total evaluation time of 128 min of walking. During data-driven optimization, each control law was evaluated for 30 s, sufficient to obtain an accurate estimate of participant motions, which were nearly steady following the rapid adaptation phase^23^. This required a total evaluation time of 32 min of walking. For each participant, the parameters identified using data-driven and metabolic approaches were similar. For example, optimized peak torque values were well correlated across methods (*R*^2^ = 0.76, *P* = 1.4 × 10^−4^, *n* = 9). On the second day, participants performed a standing rest condition followed by assistance conditions including Normal Shoes and walking with the exoskeletons under Zero Torque, Generic assistance, Metabolic Optimized assistance and Data-driven Optimized assistance. The assistance conditions for these validation tests were randomized and presented in a double-reversal order, as ABCDDCBA, to mitigate the effects of trial order related to within-day adaptation and fatigue. Each condition lasted for 6 min and included metabolic measurements.

The second experiment was used to evaluate the same set of assistance conditions at additional speeds and treadmill grades. A subset of healthy adult participants from the first experiment (*n* = 3, 3 men; age, 24.0 ± 2.0 yr; body mass, 66.0 ± 8.0 kg; height, 1.76 ± 0.05 m) completed the experiment. Participants completed the same experimental protocol used in the first tethered exoskeleton experiment for three additional walking conditions: walking at a slow speed of 0.75 m s^−1^, a fast speed of 1.75 m s^−1^, and on a 10° incline at 1.25 m s^−1^.

### Speed-adaptive controller and validation experiment

We developed a speed-adaptive controller that adjusted exoskeleton assistance based on walking speed. During real-world walking, people naturally vary their speed^27^. We hypothesized that adjusting exoskeleton assistance based on walking speed would provide larger metabolic reductions than a constant pattern of assistance. We estimated the walking speed of each step using a linear model, relating measured stride durations to measured walking speeds (Extended Data Fig. 2). Walking speed estimates from each step were used to interpolate exoskeleton assistance parameters from those optimized at a range of fixed speeds (Fig. 3a).

During speed-adaptive control, walking speed from one step was used to select the assistance parameters for the following step. We expect this approach to perform well when changes in walking speed occur slowly, or when there are rapid changes in speed but they constitute a small portion of total steps, as in natural human gait. Our experimental data are consistent with the observation that most acceleration and deceleration occurs within a few steps at the start and end of each walking bout. The expected stance duration was also adjusted based on speed estimates, following an approach established in previous research^53^. In future studies, the speed-adaptive controller could be improved to deliver more effective assistance during rapid changes in gait speed by incorporating instantaneous estimates of walking speed^54,55^ and stance duration. Acceleration regimes could also be considered, with a binning approach analogous to the one used for speeds in this study, to allow optimization of assistance specific to acceleration and deceleration phases. Other approaches, such as those using phase-based control^55^ or adjusting assistance based on changes in joint kinematics rather than walking speed^56^, may be beneficial for generalizing to a large set of activities.

We conducted a third tethered exoskeleton experiment to evaluate whether adapting assistance to variations in walking speed could provide larger reductions in metabolic cost than a fixed generic assistance profile. Healthy young adults (*n* = 3, 3 men; age, 24.0 ± 2.0 yr; body mass, 66.0 ± 8.0 kg; height, 1.76 ± 0.05 m) completed the experiment. These participants had previously completed the first two tethered exoskeleton experiments, providing Data-driven Optimized parameters for walking speeds of 0.75 m s^−1^, 1.25 m s^−1^, and 1.75 m s^−1^. Participants walked on a treadmill while the speed varied sinusoidally from 0.75 m s^−1^ to 1.75 m s^−1^ with a period of 30 s. Participants completed assistance conditions including walking in Normal Shoes and walking with the exoskeletons under Zero Torque, Generic assistance (which did not change in response to changes in speed) and Speed-adaptive assistance (using the optimized control parameters previously identified for each participant). The validation tests were randomized and presented in a double-reversal ABCDDCBA order to mitigate the effects of noise in the metabolics measurements and trial order.

### Untethered exoskeleton design

The untethered exoskeleton was designed to provide the optimized assistance parameters from the tethered exoskeleton experiments under real-world conditions. The maximum peak torque magnitude for the optimized assistance during the tethered exoskeleton study was 54 Nm when walking at a moderately fast speed of 1.5 m s^−1^. The motor and power transmission elements were designed to robustly provide this level of assistance. A portable battery was selected to allow 30 min of continuous walking on a single charge. The device was designed to be lightweight to reduce the metabolic power required to carry the exoskeleton.

The untethered exoskeleton had a mass of 1.2 kg for each ankle. Many of the mechanical elements were the same as in the tethered exoskeleton, including the frame, shoe and pressure-sensor insole. New elements included the portable motor, drum-and-cable transmission, electronics, and battery (Extended Data Fig. 3). A set of computer-aided design files and a bill of materials are provided as Supplementary Data 2.

The brushless motor (AK80-9, CubeMars) contained a single stage 9:1 gear ratio and internal motor driver electronics. This gearmotor has a rated peak torque of 18 Nm, a no-load speed of 25 rad s^−1^, and a mass of 0.5 kg. We selected this motor based on simulations with a simplified model that predicted it would be capable of applying the patterns of ankle torque and velocity that corresponded to optimized assistance in the tethered exoskeleton experiments, assuming an additional 5:1 gear ratio from the drum to the heel spur.

The custom drum was machined from 7075 aluminium, with a radius of 0.020 m. A cable connected the heel spur to the motor drum. The heel spur had a maximum lever arm (the distance from the centre of the ankle joint to the rope tie-off point) of 0.115 m. The lever arm decreased as the ankle plantarflexion angle increased, with a singularity at a maximum plantarflexion angle of 55° ensuring that no ankle torque could be applied to hyperextend the ankle joint. The torque assistance profile of the exoskeleton was not impacted by changes in the lever arm because torque was measured directly at the ankle; strain gauges on the superior and inferior surfaces of the heel lever directly sensed bending moment independent of cable force. This allowed for accurate torque control without explicitly correcting for joint angle. When the motor applied torque to the drum, a force was generated in the cable, which then transmitted this force to the heel lever, creating a torque about the ankle joint of the exoskeleton. The drum-and-cable transmission had the added benefit of being backdrivable, avoiding the possibility of force spikes that can be produced by classically stiff actuators^57,58^. The cable could also be driven to a slack state to allow the person to move freely when desired, an important capability that prevents interference when not providing assistance^59^.

The untethered exoskeleton electronics consisted of a microcontroller, portable sensing elements, a motor driver integrated into the motor and a rechargeable battery. The untethered exoskeleton used a Raspberry Pi 4b microcontroller to read sensor data and perform real-time control and optimization at a rate of 200 Hz. A breakout board enabled sensors to interface with the microcontroller. A step-down voltage converter enabled the electronics to be safely powered by a portable battery. The portable sensing elements included a rotary encoder in the ankle joint that measured ankle angle and velocity, a pressure-sensing insole in the shoe, a set of strain gauges in a full Wheatstone bridge configuration applied to the heel spur to measure torque, and an amplifier (IAA100, Futek) to allow measurement of strain-gauge signals. The pressure-sensing insole had pressure sensors located at the heel, fifth metatarsal, distal phalanx of the great toe and the first metatarsal. Fusing information from these different sensors enabled robust estimation of stance and stride period while providing measurements to extract information for optimizing assistance. This choice of sensors was guided by the design heuristic that multiple modes of sensing are important for effective exoskeleton control^60^. Muscle electrical activity could have provided additional information for control, but with the added challenge of handling noise from sensors placed on the skin^61^. The total weight of electronics was 0.15 kg.

The entire system was powered by a lithium polymer battery with a nominal voltage of 24 V, a capacity of 1,300 mAh, and a weight of 0.3 kg. Battery life was experimentally evaluated under the most demanding assistance pattern, characterized by a peak torque of 54 Nm and late timing of peak torque. Tests were conducted while walking on a treadmill at a speed of 1.5 m s^−1^. The battery was initially charged to a maximum voltage of 25.2 V and the battery life experiment was stopped once the battery voltage reached 21.6 V, corresponding to a cell voltage of 3.6 V, the minimum safe level recommended for discharging a lithium polymer battery. During testing, cell voltage was monitored by a safety regulator and an audio alarm was played once the cell voltage reached 3.6 V. We found that the 0.3-kg battery used in real-world tests allowed 36.3 min of operation under these conditions.

The design of the untethered device was guided by previous laboratory-based ankle exoskeletons, incorporating design elements that allowed for large assistive torques while maintaining comfortable forces on the body^43^. The shoe, carbon fibre struts and calf spacers were designed to be interchangeable to fit different participants, following best practices for fitting^62^. The motor-and-drum transmission and heel spur were designed to be one size fits all, with interchangeable shoes and spacers accommodating differences in foot size and mediolateral dimensions of participants’ legs. It might at first appear that the force applied by the cable between the drum and heel spur would pull the exoskeleton down the leg, but the rigid exoskeleton frame allows the axial component of this force to be reacted out at the exoskeleton joint rather than as shear on the person’s skin^43^. Thus, only a normal force is applied to the shank of the leg, which allows for more comfortable application of high torques^63^. The carbon fibre frame of the exoskeleton used stiff material and a cross-section with a high-area moment of inertia to prevent meaningful deflection during loading. As the system regulated exoskeleton joint torque, rather than motor current or velocity, and as torque was measured directly at the joint, compliance and dissipation in the transmission, exoskeleton frame and human–exoskeleton interface did not affect the accuracy or consistency of the applied torque.

The design of the untethered exoskeleton required several trade-offs. The highest design priority was providing a peak torque of 54 Nm during walking at 1.5 m s^−1^, specified from previous optimization experiments, with the least mass possible. We considered several factors to ensure that the motor would provide 54 Nm during operation. We simulated the torque needed to provide the desired assistance, overcome transmission inefficiencies, and accelerate the mass of the motor rotor and drum as required to track ankle movements during walking at 1.5 m s^−1^. The motor had to operate at a safe steady-state temperature to prevent damage to the windings. A brushless motor was selected for its relatively high efficiency and peak torque. This untethered exoskeleton was designed for the optimized parameters of our experimental participant group, and other participants may require a different device with different balance between torque and weight to provide the same reductions in the metabolic cost of walking.

Another important decision was whether to place the motor and electronics near the assisted joint or closer to the torso. The energy cost of carrying mass at distal joints is high^33^, suggesting a relocated drive approach with heavy motors carried more proximal to the centre of mass of the body. We considered mounting the motor and electronics at the hip and using a Bowden cable to transmit forces to the ankle joint. Bowden cables have an inner cable that moves relative to an outer conduit like a bicycle brake. This introduces complex transmission dynamics, including stick–slip friction, history dependence and a dependence on leg posture, making torque control more challenging, reducing control bandwidth and decreasing energy efficiency. The cables and additional electrical wires also add to the weight of the system. For these reasons, we selected a drum-and-cable transmission located on the shank of the leg. Locating motors and electronics near the assisted joint resulted in more efficient power transmission, lower transmission compliance, better control bandwidth and less total weight.

Our untethered exoskeleton was designed to allow tests of real-world personalization and resulting mobility benefits during naturalistic walking in a community setting. A significant amount of additional engineering would be required to make this device ready for everyday use by consumers. Everyday use would require easier donning and doffing, a more comfortable interface, more robust electronics hardware and more intuitive, independent control, for example, utilizing a smartphone app. In addition, the exoskeleton would have to be tested to ensure functionality during additional common activities such as navigating stairs, and to ensure that it did not interfere with common activities such as sitting and driving. While we did not directly evaluate descending stairs in this study, we did notice that the long heel spur required participants to walk carefully to avoid hitting the previous step. This design choice was made for convenience, allowing us to use as many elements from our previous tethered exoskeleton design as possible. A less obtrusive transmission would be needed for a consumer device. The commercially available Dephy ExoBoot^64^ provides an example of a more streamlined design; it has no spur behind the heel of the shoe, has simple donning and doffing features, and has minimal structure on the medial side of the leg, making it a good candidate for extended use in a large range of activities. Other autonomous ankle exoskeletons^10,17^ demonstrate complementary ways of designing hardware that is more compatible with everyday use. With increased torque capacity, more accurate torque control and real-world personalization using the approach described here, we expect commercial devices could achieve similar reductions in metabolic rate.

### Opportunistic optimization approach

We overcame the challenges of optimizing assistance during short bouts of walking at varying speeds by opportunistically accumulating data across many bouts and binning by speed. This opportunistic optimization approach used the same data-driven classification model and optimization method that were validated in the tethered experiments, with the addition of a check that sufficient consecutive steps had been collected for each control law and a method for addressing a wide range of speeds (Extended Data Fig. 4).

The opportunistic optimization method checked that sufficient steps had been collected before moving on to the next control law. We chose the requirement of 44 steps to approximate the durations used in the tethered data-driven optimization experiments. If sufficient continuous steps were not collected before the end of the walking bout, the optimizer would start over with the same controller on the next bout. Once sufficient strides were collected, the next control law was applied for that speed bin. As with the tethered experiments, the first six strides of data were discarded to avoid confounds related to rapid adaptation to a new exoskeleton control law.

The same data-driven classification model used in the tethered exoskeleton experiments was used for the real-world optimization, but a different set of assistance torque parameters were optimized. The torque parameters for peak time and fall time were fixed to the average values of the Data-driven Optimized parameters from the first tethered exoskeleton experiment (54.6% of the gait cycle and 10.0% of the gait cycle). We fixed the values of peak time and fall time because the optimized values changed little across speeds and participants, indicating that fixed values may be sufficient. The optimized values of peak torque and rise time varied substantially across speeds and participants, and so these parameters were optimized in untethered exoskeleton experiments. Optimizing two, rather than four, torque parameters reduced the dimensionality of the optimization, requiring only six, rather than eight, control laws to be collected for each generation of optimization. Reducing the number of control laws to be evaluated per generation allowed for more generations to be completed within a set experiment time, providing more frequent optimization updates and a better estimate of the optimal values. This may have come at the cost of suboptimal assistance timing parameters for some participants.

Once data for all the control laws in a generation were collected, the data-driven classification model ranked the control laws. The optimizer used this ranking to update its estimate of the optimal parameters and to adjust internal parameters, such as the convergence parameter (*σ*) that set the spread of the distribution from which to draw parameters for the next generation. Optimizations were performed for three bins of walking speed: less than 1.22 m s^−1^, between 1.22 m s^−1^ and 1.38 m s^−1^, and greater than 1.38 m s^−1^. These speeds were chosen based on the 33rd and 66th percentile of real-world walking speed distributions^27^, resulting in an equal expected likelihood for the participant to walk in each bin. Speed-adaptive control interpolated assistance based on the speed of each individual step (Extended Data Fig. 2). When a sufficient number of steps were collected for one control law, the estimated walking speeds for all steps during that control law were averaged, the corresponding speed bin was selected, and data were stored for the optimization process. When a complete generation of control laws were collected for a speed bin, control laws for that bin were ranked and the optimization parameters for that bin were updated. The estimate of the optimal assistance parameters for the other speed bins were also adjusted by a lesser amount, with the magnitude of the adjustment being proportional to the value of the convergence parameter, *σ*, for that bin (Extended Data Fig. 4). This allowed parameters in all speed bins to update more quickly at the beginning of the optimization, with decreased across-speed influences as the optimizations within each speed bin converged.

We chose to optimize a set of assistance parameters for each of three bins of walking speed, but it is possible to formulate this optimization in different ways. The data-driven classifier requires comparisons of control laws at similar walking speeds. A larger number of bins of walking speeds could be used to provide more granular speed-based adaptation, at the expense of additional time to optimize a larger number of assistance parameters. It may also be possible to simultaneously solve for a larger set of control parameters that fully define the speed-adaptive controller, but this would introduce challenges related to the larger parameter space, interaction effects between parameters, and poorly conditioned maps between parameters that have a strong effect on assistance at one speed and little effect on assistance at different speeds. Instead, we opted for a small set of speed bins, with a relatively simple approach to updating the optimal parameter estimates.

### Real-world optimization experiments

In the real-world optimization experiments, we used the untethered exoskeleton to optimize assistance during naturalistic bouts of walking and then evaluated the optimized assistance profiles under real-world and treadmill conditions.

Healthy adult participants (*n* = 10, 6 men and 4 women; age, 24.2 ± 1.8 yr; body mass, 67.0 ± 8.2 kg; height, 1.72 ± 0.07 m) completed a two-day protocol. On the first day, participants walked outside in a public setting along a path consisting of concrete, asphalt and brick sidewalks (Fig. 5b) for approximately 1 h while the untethered exoskeleton provided assistance and performed data-driven optimization. To emulate natural walking, the participants received audio cues to tell them to start and stop walking bouts. The durations of these bouts were randomly drawn from a preselected distribution (Fig. 5d) that matched naturally occurring bout durations^34^. Participants stood at rest between bouts for a randomized duration of 5 s to 10 s. To encourage a normal range of speeds, we provided participants with audio prompts, such as “Walk as if you were walking to catch a bus” and “Walk as if you were walking a small dog”, at the start of each bout. A previous study^28^ demonstrated that these prompts were associated with different self-selected walking speeds, and we expected that participants would adopt similar speeds. We randomly sampled from a distribution of speeds (Fig. 5c) that mimicked natural walking patterns measured in a previous study^27^.

On the second day, participants performed outdoor and treadmill validation tests to evaluate the benefits provided by Real-world Optimized assistance. For the outdoor validation, participants walked along a 566-m path in the same public setting with a fixed ordering of bouts of specific distances and corresponding speed prompt commands that were selected to match real-world distributions^27,34^. Distances were set using cones to mark stopping locations, which ensured consistent distances for each bout. Participants completed this outdoor course once for each condition, including Real-world Optimized assistance, Generic Speed-adaptive assistance and Normal Shoes. The ordering of the conditions was randomized to minimize effects of testing order (Extended Data Table 1). The double-reversal protocol, used in the first three laboratory experiments, was not used because the outdoor experiments took significantly more time owing to the longer trial time, varying self-selected walking speeds, short bouts of walking, and rest periods between bouts and conditions. Each real-world condition required about 15 min, compared with about 8 min for each treadmill condition. Outdoor and indoor tests of Real-world Optimized assistance were conducted on the same day to avoid confounding effects from differing respirometry system calibrations. The total walking time for these two experiments was about 1.5 h, and we found that participants were not able to complete the additional 1.5 h of walking that would have been required for a double-reversal approach without experiencing fatigue. For the 3 min following completion of the path, participants stood at rest while respirometry data were collected to capture the total metabolic cost of completing the course. The duration of walking for each bout was timed with a stopwatch. Walking speed for each bout was computed by dividing the fixed distance for that bout by the time spent walking during that bout. Walking speed for each condition was calculated as the total distance travelled divided by the total time spent walking while navigating the course.

The indoor validation consisted of a standing rest condition followed by six treadmill conditions, each lasting 6 min. Participants walked on a treadmill at 1.25 m s^−1^, at 1.5 m s^−1^, and on an incline of 10° at 1.25 m s^−1^. Participants completed each treadmill speed and grade twice, once with Real-world Optimized assistance, as identified during the outdoor optimization period, and once with Normal Shoes. The ordering of conditions was randomized, with a constraint that the exoskeleton would only be donned and doffed one time to reduce experiment time (Extended Data Table 2). We did not use the double-reversal protocol in these tests because we found that participants could not reliably complete the additional 1.5 h of walking that would have been required without experiencing fatigue, and so instead used the more typical approach of single presentations with randomized order.

One pilot participant completed additional indoor conditions, walking at 1.25 m s^−1^ with an incline of 5°, walking at 1.25 m s^−1^ with a load of 20% of their body weight carried in a weight vest, and stair climbing on a stairmill at 50 steps per minute. The results (Extended Data Fig. 6) were used to test the generality of the approach. Owing to the small sample size (*n* = 1), this figure and the numerical results for change in metabolic rate are not included in the main text.

We performed a naturalistic overground experiment in an outdoor, suburban community setting. People require assistance in many different settings and for a variety of additional activities, and future work should extend the approaches presented in this study to optimize assistance and evaluate assistive device benefits for a wider range of tasks. For example, future devices could sense, adapt to and optimize assistance for various grades^55^, during stair navigation^17^ and over rough terrain^54^. These future studies will provide additional translational impact for daily mobility.

### Comparison with other untethered exoskeletons

We compared the benefits of Real-world Optimized assistance with the untethered exoskeleton to the best results of comparable previous studies^10–16^. To allow direct comparison, we considered only studies that tested untethered devices, report data for normal walking, tested similar walking conditions, tested sufficient participants and used standard data-processing techniques. For untethered exoskeletons, the most relevant outcome is the percent change in the energy cost of walking with exoskeleton assistance to walking in normal shoes without the exoskeleton. Changes in walking conditions can affect outcomes, so we considered studies conducted at within 10% of the speeds and inclines that we tested. Before conducting our final experiment, we selected the speeds (1.25 m s^−1^ and 1.5 m s^−1^) and inclines (10°) that captured the largest percent reductions in metabolic rate that had previously been observed for any exoskeleton study in the literature. We compared with previous exoskeleton studies with at least five participants, because studies reporting data from fewer tests are difficult to interpret owing to measurement noise and inter-participant variability. We compared with previous studies in which the metabolic cost of walking was calculated using standard techniques, by averaging respirometry measurements during the last 2 min or 3 min of a 5-min or 6-min steady-state treadmill condition. One previous exoskeleton study^65^ was excluded because steady-state metabolic cost was computed by taking the median of respirometry measurements. We found that using the median rather than the mean to compute metabolic rate in our untethered exoskeleton study increased the magnitude of the reductions in metabolic cost by an average of 7% across participants. This is a large amount compared with the total improvement of 23%, indicating that the median and mean measurements are not equivalent. We were not able to obtain the data from the previous study that would have allowed computation of the mean percent change in metabolic rate.

To keep Extended Data Fig. 5 legible, we only depict studies reporting results within a 5% change in metabolic rate of the best previous value for that condition category. There are several other untethered exoskeletons that have provided some reduction in metabolic rate under conditions similar to those tested in this study. For example, the Dephy ExoBoot, the commercially available exoskeleton with the most similar features to the prototype tested in this study, can provide a 5.2% reduction in metabolic energy consumption compared with walking with Normal Shoes while walking on a treadmill with time-varying speed^64^. Another technologically mature untethered exoskeleton, the MyoSuit Beta, has shown that hip assistance during outdoor uphill walking can reduce metabolic rate compared with wearing the exoskeleton in Zero Torque mode^37^. Sufficient data are not yet available to estimate the benefits compared with walking without the exoskeleton. In the interests of clarity, we did not include the results of all previous exoskeleton experiments in Extended Data Fig. 5.

We compared the results of this study against all types of lower-limb exoskeleton, including devices that assist the knees and hips, to provide the clearest understanding of the relative benefits of this design and personalization approach. Considering instead only ankle exoskeletons would allow for a more mechanistic comparison of system components and biomechanics outcomes, at the cost of reduced generality of the high-level findings. As exoskeleton technologies mature and address more tasks and populations, joint-specific benefits or restrictions related to specific conditions may make it more sensible to apply joint-specific comparisons in some contexts.

Our untethered exoskeleton provided the largest reductions in the metabolic cost of walking primarily owing to the way it personalized assistance to individual users, but hardware design differences may also have contributed to its efficacy. Design differences between the untethered exoskeleton and some previous devices include: directly measuring joint torque, rather than inferring it from motor current; providing slack in the transmission to avoid interference during leg swing and Zero Torque mode; and larger peak torque capabilities, such that benefits were limited more by the user’s ability to accept assistance than by limitations in the hardware. Directly measuring joint torque requires additional electronics hardware for sensing and signal processing but enables more precise control of applied torques, which eliminates errors owing to model mismatch and power losses in the transmission and interface with the body. This helps provide users with a consistent assistance pattern. Placing slack in the transmission cable during periods when zero torque is desired prevents the inadvertent application of the small damping torques needed for linear feedback control. Although they may seem small, these damping torques can substantially increase user effort. Allowing for larger peak torques, in this case approximately twice the value of previous untethered ankle exoskeletons^10,17,64,66^, allowed for a larger range of possible assistance parameters. This makes it more likely that the global optimum for a given participant and walking speed lie within the range of hardware-feasible control. Larger torques require a rigid frame to react out transmission forces in the exoskeleton joint^43^, rather than through shear on the skin^63^, to maintain user comfort. The present results would therefore seem to favour devices that can apply higher torques to achieve greater benefits from assistance, at the cost of greater worn mass. This relationship, however, will be sensitive to the populations and tasks that are assisted. The above design decisions enabled the untethered ankle exoskeleton in this study to provide accurate, reliable and substantial assistance to the user, which enabled participants to obtain large net benefits from real-world personalized assistance.

### Participant surveys on exoskeleton usability

Participants completed a series of surveys to evaluate the ease of use, comfort and functionality of the untethered exoskeleton after completion of all the experiments. Participants completed a System Usability Scale survey^67^ to determine how easy it was to operate the untethered exoskeleton. Users reported that the exoskeleton was relatively easy to use, with an overall score of 72.5 (Extended Data Table 3), placing it in the 65th percentile of 5,000 devices previously surveyed^42^. Participants also completed surveys adapted from the Orthotics and Prosthetics Users’ Survey^68^, which acts as a self-report instrument for evaluating the outcomes of prosthetics and orthotics services in a clinically useful manner. Among comfort-related outcomes, participants were most likely to agree that the weight of the device was manageable, that it was easy to put on and that their clothes were free of wear (Extended Data Table 4). Participants were more likely to be neutral or to disagree that the exoskeleton would be comfortable throughout the day. Among outcomes related to functionality, participants found standing, walking indoors and outdoors, and donning and doffing the exoskeleton to be easy or very easy (Extended Data Table 5). Participants found picking objects up from the ground and walking up steep ramps to be slightly difficult. When asked whether they would prefer to use the exoskeleton or normal shoes if they had to complete the outdoor walking course again, six out of the ten participants reported that they would prefer to use the exoskeleton.

### Reporting summary

Further information on research design is available in the Nature Research Reporting Summary linked to this article.

## Online content

Any methods, additional references, Nature Research reporting summaries, source data, extended data, supplementary information, acknowledgements, peer review information; details of author contributions and competing interests; and statements of data and code availability are available at 10.1038/s41586-022-05191-1.

### Supplementary information

Reporting Summary

Supplementary Data 1 Sample code consisting of functions used with the opportunistic optimization approach. This simulates an optimization of the exoskeleton torque parameters over multiple generations. This code uses Python version 3.6.1. The required python packages are numpy (1.17.4), scikit-learn (0.21.3), scipy (1.3.2) and matplotlib (2.0.2).

Supplementary Data 2 The computer-aided design files and bill of materials needed to render and replicate the untethered exoskeleton. These computer-aided design files depict the individual hardware elements of the untethered exoskeleton and a complete assembled structure. These files can be used to manufacture and assemble a replica of the system for full reproduction of our untethered ankle exoskeleton.

Supplementary Video 1 A close-up of the untethered exoskeleton assisting a person walking in a public setting. The video is slowed down by a factor of four to allow better visualization of the motor-and-drum transmission applying torque about the ankle joint to assist the person as they extend their ankle and push off of the ground with their toes.

Supplementary Video 2 Real-world optimization of exoskeleton assistance during one hour of walking. Every 44 steps, a new set of assistance parameters were provided to the person. This allowed the optimizer to quickly evaluate many possible assistance parameters. Audio cues provided to the participant through an earpiece prompted them to self-select a naturalistic range of walking speeds. The prompts for each bout of walking are shown as text. Participants also received audio cues to stop walking, the timing of which were chosen to produce a naturalistic distribution of bout durations. Playback is at 15 times the actual speed.

Supplementary Video 3 Validation of Real-world Optimized exoskeleton assistance. The video shows 7 of the 15 outdoor walking bouts used to determine the real-world benefits exoskeleton assistance. Participants completed all walking bouts under three walking conditions: Real-world Optimized exoskeleton assistance, Generic Speed-adaptive exoskeleton assistance and Normal Shoes. Participants walked between yellow cones whose separations were chosen to provide a natural distribution of bout lengths. Different verbal prompts were given to the participant to elicit a naturalistic range of walking speeds. A portable respirometry system measured the ground-truth energy expenditure during movement. The humming of the respirometry air sampling system is audible in the video.

### Source data

Source Data Figs. 2–5 and Extended Data Figs. 1, 5 and 6

## Figures and Tables

**Extended Data Table 1:**
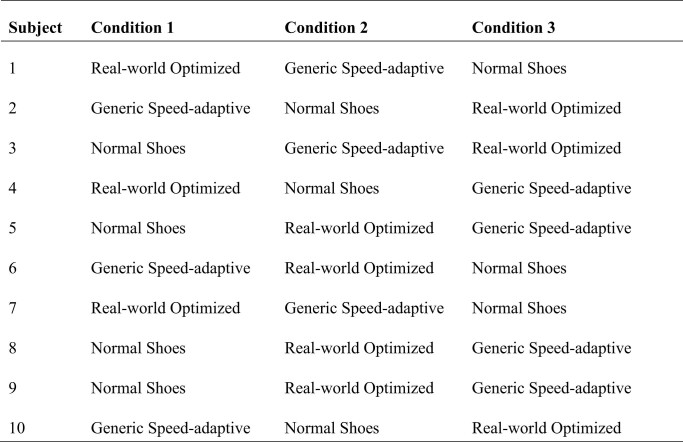
Randomized condition ordering for the outdoor evaluations in the real-world exoskeleton optimization experiment

Participants completed a quiet standing condition followed by Normal Shoes, Generic Speed-adaptive exoskeleton assistance, and Real-world Optimized exoskeleton assistance conditions, presented in random order.

**Extended Data Table 2:**
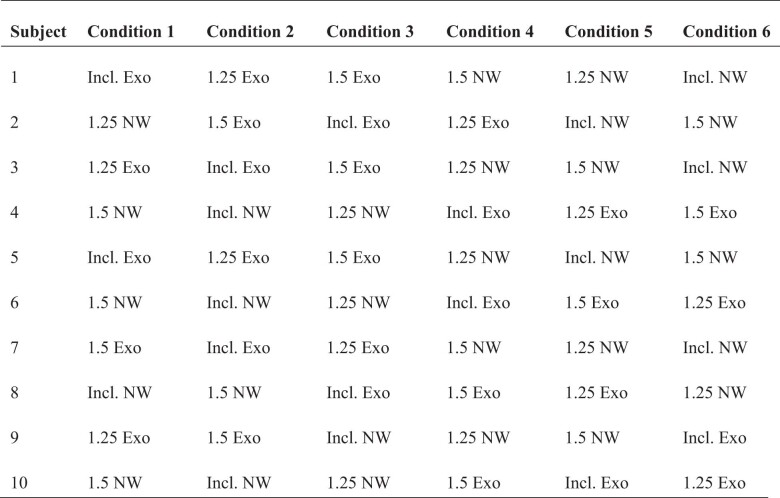
Randomized condition ordering for the indoor evaluations in the real-world exoskeleton optimization experiment

The labels ‘1.25’, ‘1.5’, and ‘Incl.’ represent the treadmill conditions of walking at 1.25 m s^−1^, walking at 1.5 m s^−1^, and walking on a 10° incline at 1.25 m s^−1^. The labels ‘Exo’ and ‘NW’ represent the Real-world Optimized assistance and Normal Shoes conditions. Conditions were presented in random order, with a constraint that the exoskeleton would only be donned and doffed once to reduce experiment time.

**Extended Data Table 3:**
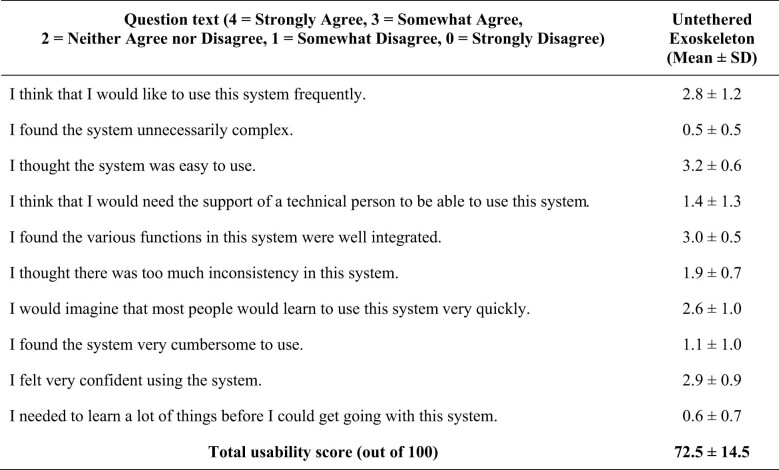
Usability survey results for exoskeleton participants

The System Usability Scale^67^, which uses a Likert scale, was used to evaluate the usability of the untethered exoskeleton. Participants (*n* = 10) completed this survey after completing all walking experiments. The untethered exoskeleton was in the 65th percentile of a distribution of 5000 devices previously evaluated with the System Usability Scale^42^.

**Extended Data Table 4:**
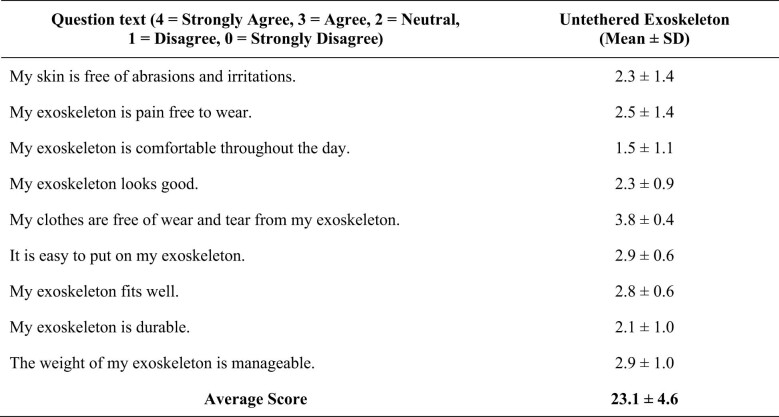
Survey results on the comfort of the untethered exoskeleton

This survey was adapted from the Orthotics and Prosthetics Users’ Survey^68^, which acts as a self-report instrument for evaluating clinically useful outcomes of prosthetics and orthotics services. Participants (*n* = 10) completed the survey after completing all walking experiments.

**Extended Data Table 5:**
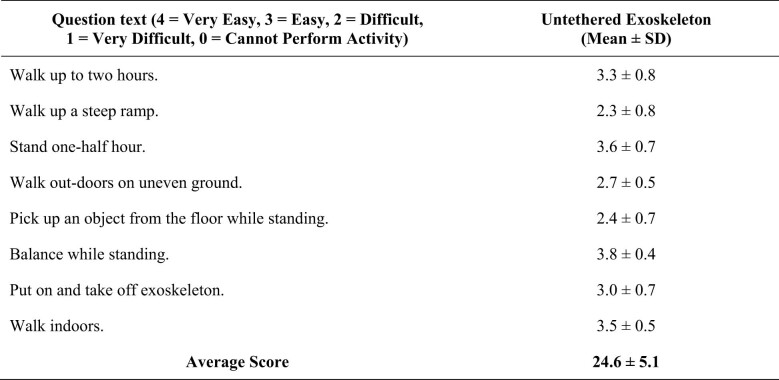
Survey results on the functionality of the untethered exoskeleton

This survey was adapted from the Orthotics and Prosthetics Users’ Survey^68^, which acts as a self-report instrument for evaluating the outcomes of prosthetics and orthotics services in a clinically useful manner. Participants (*n* = 10) completed this survey after completing all walking experiments.

## Data Availability

All study data necessary to replicate this work are available in the Source Data included with the paper. Computer-aided design files and a bill of materials for the untethered ankle exoskeleton are provided in Supplementary Data 2. Source data are provided with this paper.

## Code availability

Optimization code samples are provided in Supplementary Data 1. This code uses Python version 3.6.1. The required python packages are numpy (1.17.4), scikit-learn (0.21.3), scipy (1.3.2) and matplotlib (2.0.2).
