# Supplementary material for: Personalizing exoskeleton assistance while walking in the real world
Source: Nature. 2022 Oct 12;610(7931):277–82. doi: 10.1038/s41586-022-05191-1 (PMC9556303; doi:10.1038/s41586-022-05191-1)
Supplement: Supplementary file 1 — Reporting Summary [file 41586_2022_5191_MOESM1_ESM.pdf]

nature research

Corresponding author(s): Patrick Slade

Last updated by author(s): May 24, 2022

Reporting Summary

Nature Research wishes to improve the reproducibility of the work that we publish. This form provides structure for consistency and transparency in reporting. For further information on Nature Research policies, see our Editorial Policies and the Editorial Policy Checklist.

Statistics

For all statistical analyses, confirm that the following items are present in the figure legend, table legend, main text, or Methods section.

n/a	Confirmed	
☐	☒	The exact sample size (*n*) for each experimental group/condition, given as a discrete number and unit of measurement
☐	☒	A statement on whether measurements were taken from distinct samples or whether the same sample was measured repeatedly
☐	☒	The statistical test(s) used AND whether they are one- or two-sided<br>*Only common tests should be described solely by name; describe more complex techniques in the Methods section.*
☒	☐	A description of all covariates tested
☐	☒	A description of any assumptions or corrections, such as tests of normality and adjustment for multiple comparisons
☐	☒	A full description of the statistical parameters including central tendency (e.g. means) or other basic estimates (e.g. regression coefficient) AND variation (e.g. standard deviation) or associated estimates of uncertainty (e.g. confidence intervals)
☐	☒	For null hypothesis testing, the test statistic (e.g. F, t, r) with confidence intervals, effect sizes, degrees of freedom and P value noted<br>*Give P values as exact values whenever suitable.*
☒	☐	For Bayesian analysis, information on the choice of priors and Markov chain Monte Carlo settings
☒	☐	For hierarchical and complex designs, identification of the appropriate level for tests and full reporting of outcomes
☒	☐	Estimates of effect sizes (e.g. Cohen's *d*, Pearson's *r*), indicating how they were calculated

Our web collection on statistics for biologists contains articles on many of the points above.

Software and code

Policy information about availability of computer code

Data collection	Data collection was performed using Python version 3.6.1. The required python packages are numpy (1.17.4), scikit-learn (0.21.3), scipy (1.3.2), matplotlib (2.0.2). Software scripts used for data collection are available in the Supplementary Data S2 included with the manuscript.
Data analysis	Data analysis was performed using Python version 3.6.1. The required python packages are numpy (1.17.4), scikit-learn (0.21.3), scipy (1.3.2), matplotlib (2.0.2). Software scripts used for data analysis are available in the Supplementary Data S2 included with the manuscript.

For manuscripts utilizing custom algorithms or software that are central to the research but not yet described in published literature, software must be made available to editors and reviewers. We strongly encourage code deposition in a community repository (e.g. GitHub). See the Nature Research guidelines for submitting code & software for further information.

Data

Policy information about availability of data

All manuscripts must include a data availability statement. This statement should provide the following information, where applicable:

- Accession codes, unique identifiers, or web links for publicly available datasets
- A list of figures that have associated raw data
- A description of any restrictions on data availability

All data necessary to replicate this work are available in the Supplementary Data included with the Manuscript. The data to replicate all figures is included in Supplementary Data S1. The code to collect and analyze the data is included in Supplementary Data S2. The hardware files, assembly, and bill of materials to replicate the untethered exoskeleton is included in Supplementary Data S3.

Field-specific reporting

Please select the one below that is the best fit for your research. If you are not sure, read the appropriate sections before making your selection.

☒ Life sciences ☐ Behavioural & social sciences ☐ Ecological, evolutionary & environmental sciences

For a reference copy of the document with all sections, see nature.com/documents/nr-reporting-summary-flat.pdf

Life sciences study design

All studies must disclose on these points even when the disclosure is negative.

Sample size	We conducted a power analysis based on previous lab-based optimization experiments and found that a sample size of eight participants was necessary for the planned validation experiments. This analysis used a power of 1 - β = 0.8, a significance level of α = 0.05, the difference in mean metabolic rate between optimized (1.44 W/kg) and generic (1.64 W/kg) assistance from a prior experiment (Poggensee and Collins, 2021), and the variability in metabolic rate (standard deviation = 0.15 W/kg) from the same experiment. We collected data from nine participants for the tethered exoskeleton experiments and ten participants for the untethered exoskeleton experiments, with additional participants protecting against data loss due to sensor failure. All participants that were recruited completed the corresponding experiment, and data from all participants were included in each corresponding analysis.
Data exclusions	No exclusions were made.
Replication	We have made the experimental data available and provided all code used to replicate the results shown in the Figures.
Randomization	All participants completed the same set of experimental conditions which were in a randomized order.
Blinding	All subjects were in the same group during the experiment, so the blinding was not relevant to the study.

Reporting for specific materials, systems and methods

We require information from authors about some types of materials, experimental systems and methods used in many studies. Here, indicate whether each material, system or method listed is relevant to your study. If you are not sure if a list item applies to your research, read the appropriate section before selecting a response.

Materials & experimental systems

n/a	Involved in the study	
☒	☐	Antibodies
☒	☐	Eukaryotic cell lines
☒	☐	Palaeontology and archaeology
☒	☐	Animals and other organisms
☐	☒	Human research participants
☒	☐	Clinical data
☒	☐	Dual use research of concern

Methods

n/a	Involved in the study	
☒	☐	ChIP-seq
☒	☐	Flow cytometry
☒	☐	MRI-based neuroimaging

Human research participants

Policy information about studies involving human research participants

Population characteristics	The experiment that validated the benefits of assistance from an untethered ankle exoskeleton was from a population of adults with characteristics: n = 10, 6 men and 4 women; age = 24.2±1.8yr; body mass = 67.0±8.2kg; height = 1.72±0.07m.
Recruitment	Healthy adult participants were recruited when they had no ongoing or previous leg injury in the past 6 months. All participants were volunteers that provided written and informed consent before completing the protocol. Participants that had at least 8 hours of previous experience walking in powered ankle exoskeletons were recruited to avoid complexities due to learning to use the device. This restricted participants to people that were familiar to the exoskeletons, and possibly biased towards using technology, which may have impacted survey results.
Ethics oversight	Stanford University Institutional Review Board (IRB-48749)

Note that full information on the approval of the study protocol must also be provided in the manuscript.
